# Identification of three hub genes related to the prognosis of idiopathic pulmonary fibrosis using bioinformatics analysis

**DOI:** 10.7150/ijms.73305

**Published:** 2022-08-15

**Authors:** Enze Wang, Yue Wang, Sijing Zhou, Xingyuan Xia, Rui Han, Guanghe Fei, Daxiong Zeng, Ran Wang

**Affiliations:** 1 Department of respiratory and critical care medicine, the first affiliated hospital of Anhui medical university, Hefei 230022, China.; 2 Department of Infectious Diseases, Hefei second people's hospital, Hefei 230001, China.; 3 Department of occupational medicine, Hefei third clinical college of Anhui Medical University, Hefei 230022, China.; 4 Department of pulmonary and critical care medicine, Suzhou Dushu Lake Hospital, Suzhou, 215006, China.; 5 Department of pulmonary and critical care medicine, Dushu Lake Hospital Affiliated to Soochow University, Medical Center of Soochow University, Suzhou, 215006, China.

**Keywords:** IPF, Prognosis, Bronchoalveolar lavage cells, Genes, Bioinformatics

## Abstract

**Background:** Idiopathic pulmonary fibrosis (IPF) is a chronic respiratory disease characterized by peripheral distribution of bilateral pulmonary fibrosis that is more pronounced at the base. IPF has a short median survival time and a poor prognosis. Therefore, it is necessary to identify effective prognostic indicators to guide the treatment of patients with IPF.

**Methods:** We downloaded microarray data of bronchoalveolar lavage cells from the Gene Expression Omnibus (GEO), containing 176 IPF patients and 20 controls. The top 5,000 genes in the median absolute deviation were classified into different color modules using weighted gene co-expression network analysis (WGCNA), and the modules significantly associated with both survival time and survival status were identified as prognostic modules. We used Lasso Cox regression and multivariate Cox regression to search for hub genes related to prognosis from the differentially expressed genes (DEGs) in the prognostic modules and constructed a risk model and nomogram accordingly. Moreover, based on the risk model, we divided IPF patients into high-risk and low-risk groups to determine the biological functions and immune cell subtypes associated with the prognosis of IPF using gene set enrichment analysis and immune cell infiltration analysis.

**Results:** A total of 153 DEGs located in the prognostic modules, three (TPST1, MRVI1, and TM4SF1) of which were eventually defined as prognostic hub genes. A risk model was constructed based on the expression levels of the three hub genes, and the accuracy of the model was evaluated using time-dependent receiver operating characteristic (ROC) curves. The areas under the curve for 1-, 2-, and 3-year survival rates were 0.862, 0.885, and 0.833, respectively. The results of enrichment analysis showed that inflammation and immune processes significantly affected the prognosis of patients with IPF. The degree of mast and natural killer (NK) cell infiltration also increases the prognostic risk of IPF.

**Conclusions:** We identified three hub genes as independent molecular markers to predict the prognosis of patients with IPF and constructed a prognostic model that may be helpful in promoting therapeutic gains for IPF patients.

## Introduction

Idiopathic pulmonary fibrosis (IPF) is a chronic and progressive interstitial lung disease (ILD) characterized by unexplained lung's scarring and extensive remodeling^1^, ^2^. Although IPF is a rare disease, its incidence is increasing annually, and it primarily affects men aged > 50 years^3^, which may be related to an aging population and increased awareness. Studies have shown that the incidence of IPF is approximately 2-30 cases per 100,000 person-years, with a prevalence of approximately 10-60 cases per 100,000 people^4^. Because of its rapid clinical progression, IPF usually has a poor long-term prognosis, with a median survival of only 2-3 years after diagnosis^5^, ^6^. Given the lack of effective molecular markers, the prognostic risk of IPF is often difficult to assess accurately. Therefore, it is necessary to develop predictive models for clinical treatment of IPF.

IPF is typically radiographically characterized by a peripheral distribution of bilateral fibrosis, more pronounced at the base, and it can be confirmed by lung biopsy when the diagnosis is uncertain^7^, ^8^. In recent years, some studies have used molecular biomarkers to assess the clinical course of IPF and achieved certain results^9^. However, most studies have used lung biopsies, which are invasive and not readily available for diagnosis. Bronchoalveolar lavage (BAL) is a technique in which sterile saline is pumped into the lungs, and the infusion is aspirated for analysis. It is a useful adjunct in the diagnostic evaluation for patients with ILD and can be used to identify infection and other inflammatory diseases^10^, ^11^. As a noninvasive and convenient detection method, BAL is more feasible for identifying molecular biomarkers from the gene expression profile of bronchoalveolar lavage cells (BLCs) to predict the prognosis of IPF.

In this study, we aimed to apply weighted gene co-expression network analysis (WGCNA)^12^ to identify biomarkers associated with IPF prognosis in BLCs. First, we downloaded mRNA expression profiles and corresponding clinical information from the Gene Expression Omnibus (GEO). Then, WGCNA and differential expression analysis were used to identify prognostic gene set. Next, enrichment analysis was conducted to identify the important biological pathways affected. Finally, we constructed and verified prognostic models and compared the different pathways between different risk groups. We believe that our results may help with the diagnosis and treatment of IPF.

## Methods

### Dataset collection and processing

**Figure 1:**
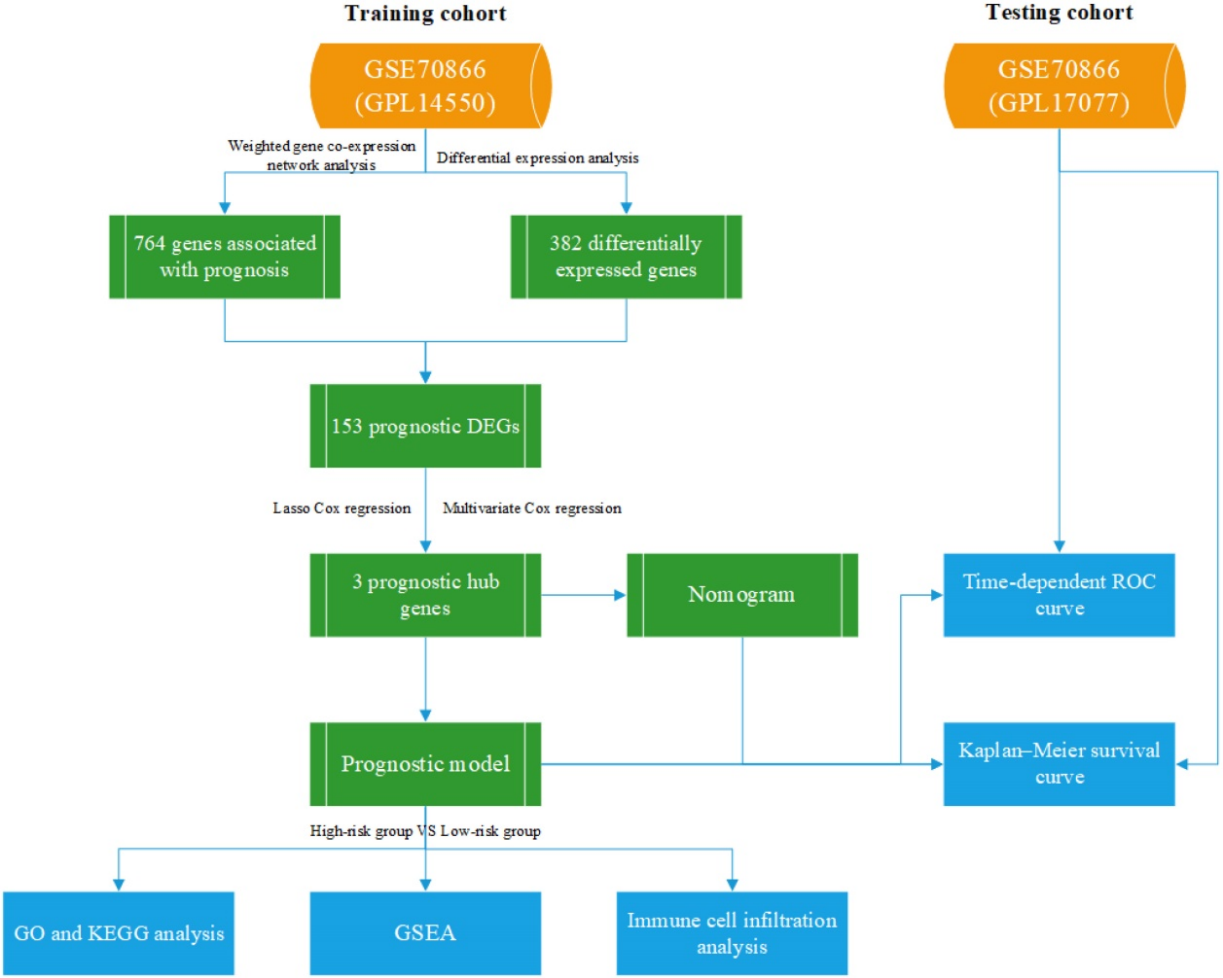
The flow chart of our study.

All data are available on the GEO dataset GSE70866, which contains 196 bronchoalveolar lavage fluid (BALF) samples consisting of 20 control individuals and 176 IPF patients^13^. Twenty control individuals and 112 IPF patients, analyzed using GPL14550, were divided into the training cohort, and the other 64 patients, analyzed using GPL17077, were divided into the testing cohort. Our study was not subject to review by the relevant ethics committee, because the information was obtained directly from a public database. Figure 1 shows the research process used in this study.

### Screening of DEGs and construction of co-expression network

Firstly, we used R package “limma” for data normalization and identification of DEGs between IPF and healthy individuals in the training cohort^14^. The absolute value of log2 fold change (FC) was greater than 1, and the adjusted p-value (adj.P) was less than 0.05, as thresholds were used to define DEGs, which contained a total of 382 genes.

**Figure 2:**
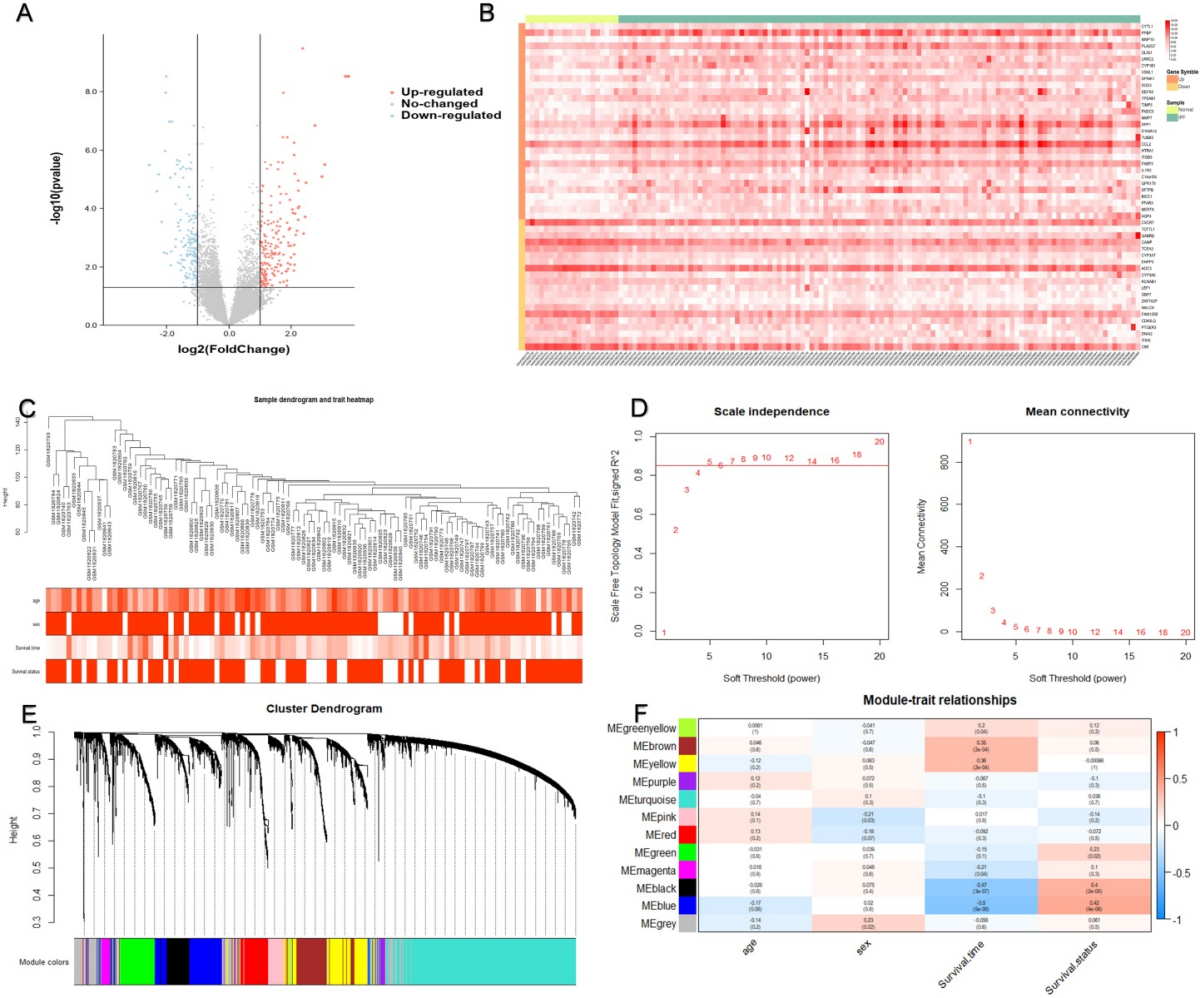
** Identification of the prognostic module using WGCNA.** (A) Differences in gene expression levels between IPF group and control group. (B) The expression of the top 30 upregulated DEGs and the top 20 downregulated DEGs in the training cohort. (C) 105 IPF samples were included in WGCNA analysis. (D) The soft thresholding power was five when 0.85 was defined as the related coefficient standard. (E) Cluster dendrogram and network heatmap for modules from WGCNA. Twelve modules were identified, with each color representing one module. (F) Black and blue modules were significantly correlated with both survival time and status. The red color represents positive correlation, and the blue color represents negative correlation. IPF, idiopathic pulmonary fibrosis; DEGs, differentially expressed genes; WGCNA, weighted gene co-expression network analysis.

Co-expression modules were constructed using via WGCNA, an analysis method to analyze gene expression patterns of multiple samples. WGCNA can cluster genes with similar expression patterns and analyze the association between modules and specific traits and phenotype. The gene expression levels of 112 IPF patients were sequenced according to the order of median absolute deviation, and the top 5000 genes were selected to construct the expression matrix. After seven outlier samples were deleted, standard scale-free network analysis was performed on the remaining 105 samples. When we defined 0.85 as the scale-free fitting index, the soft thresholding power was 5 (Figure 2D). The topological overlap matrix (TOM) was transformed by an adjacency matrix and corresponding dissimilarity (1-TOM) values were computed. We used dynamic tree cut method to identify the module. The lower limit of gene number of modules 30 and the height cut-off value 0.25 were selected as the criteria to distinguish modules. Pearson correlation analysis was used to assess the correlation between clinical characteristics (including age, sex, survival time and survival status) and gene modules. Prognostic modules were defined as modules that were significantly associated with both survival time and survival status.

### Screening of prognostic hub genes

We selected target genes from gene sets included in both prognostic modules and DEGs. Genes significantly associated with prognosis were defined as DEGs with gene significance (GS) > 0.4 and weighted correlation index of module members (MM) > 0.7. Next, we performed further analyses of these genes in 112 IPF samples from the training cohort. Lasso Cox regression was conducted to search for key genes using the R package “glmnet”^15^. Hub genes were identified using multivariate Cox regression analysis to further confirm the best prognostic DEG signature.

### Construction of prognostic model and nomogram

The risk score formula for IPF patients is as follows: 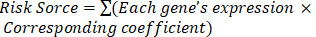. Based on the median risk score, we divided all patients into low- and high-risk groups, and time-dependent receiver operator characteristic (ROC) curves were used to test the risk score. A nomogram was constructed to predict the survival probability of IPF patients with age, sex, and hub genes using R package “rms”. The performances of the risk score and nomogram were verified in the testing cohort.

### Enrichment analysis

The Metascape database and online tools were used to identify the functional annotation of all DEGs in the OS-related module^16^. Analysis thresholds were set as minimum counts > 3, enrichment factors > 1.5, and p-value < 0.01. Then, in order to search for the potential differential expression pathways in different risk groups, the gene set enrichment analysis (GSEA) was performed from R package “clusterProfiler”^17^. We chose the c5.go.v7.4.entrez.gmt and c2.cp.kegg.v7.4.entrez.gmt (from Molecular Signatures Database) as the reference gene sets and adj.P < 0.05 as the cut-off criteria.

### Comparison of immune cell infiltration

To compare the heterogeneity of immune cell infiltration in patients with different risk groups, CIBERSORT algorithms were used to calculate the proportions of immune cell subtypes in the R platform^18^. Finally, Spearman analysis was used to evaluate the correlation between the degree of infiltration of immune cell subtypes and the expression levels of the prognostic hub genes.

### Statistical analysis

Most analyses were performed using the software R4.1.1. If not otherwise specified, a p-value < 0.05 was considered statistically significant.

## Results

### Demographic data

In the training cohort, the mean age of the 112 IPF patients was 68 years, and that of the 20 healthy subjects was 61.9 years. The mean age of the 64 patients with IPF in the testing cohort was 68.3 years. IPF was most common in men in both the training and testing cohorts (83% men patients vs 17% women in the training cohort and 80% men patients vs 20% women in the testing cohort). Table 1 presents the demographic data used in this study.

### Identification of DEGs and modules related to the prognosis

A total of 382 DEGs were identified in this dataset, which contained 208 upregulated and 174 downregulated genes. Figure 2(A) is a volcano map of all gene expressions, and Figure 2(B) shows a heatmap of the top 30 upregulated genes and the top 20 downregulated genes.

WGCNA analysis was performed on the differential gene expression profiles of 105 IPF patients with age, sex, survival time and survival status (Figure 2C). Twelve modules were identified by consensus (Figure 2E). The DEGs between the black and blue modules were most significantly associated with both survival time and survival status (Figure 2F). Therefore, we identified black and blue modules as key modules associated with prognosis, which consist of a total of 764 genes.

### Identification of prognostic hub genes

**Figure 3:**
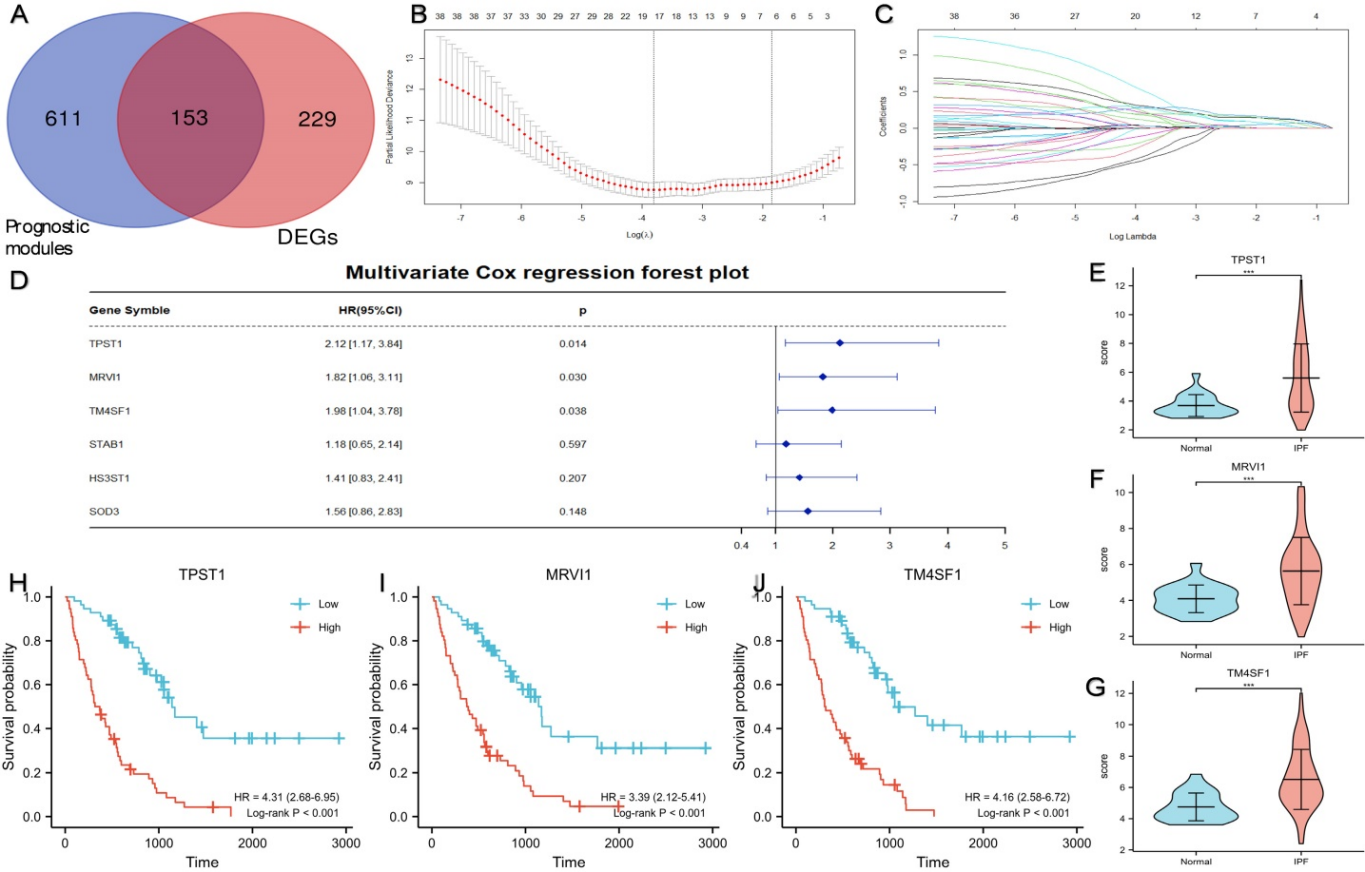
** Identification of three prognostic hub genes using Lasso Cox regression analysis and multivariate Cox regression analysis.** (A) A total of 153 DEGs located in the prognostic modules. (B) Partial likelihood deviance of the tuning parameter (λ). We chose the largest λ at which the mean square error was within one standard error of the minimal criteria, and it was represented by a dotted line on the right. (C) Lasso coefficient profiles of the 38 important genes. Different colored curves represent different genes. The upper x-axis represents the number of non-zero coefficients in the model. (D) The result of multivariate Cox regression analysis and the forest plot of four key genes related to prognosis. The expression levels of TPST1 (E), MRVI1 (F), and TM4SF1 (G) between the IPF group (n = 112) and the control group (n = 20). Kaplan-Meier survival curve of TPST1 (H), MRVI1 (I), and TM4SF1 (J) in training cohort. ***P < 0.001.

Figure 3A showed a total of 153 DEGs contained in the prognostic modules. 38 genes with GS > 0.4 and MM > 0.7 were defined as important prognostic genes in the turquoise and brown modules (Table 2). TPST1, HS3ST1, TM4SF1, SOD3, MRVI1, and STAB1 were identified as key genes by Lasso Cox regression analysis (Figure 3B-C). Finally, according to the multivariate Cox regression analysis of six key genes, TPST1, MRVI1, and TM4SF1 were identified as hub genes (Figure 3D). The violin plot showed that the expression of TPST1, MRVI1, and TM4SF1 in the IPF group was visibly higher than that in the control group (Figure 3E-G). Kaplan-Meier (KM) survival curves showed that these three genes were closely related to the prognosis of IPF. Patients with high expression of three hub genes had shorter survival times and worse prognoses than those with low expression (Figure 3H-J).

### Construction of prognostic model

**Figure 4:**
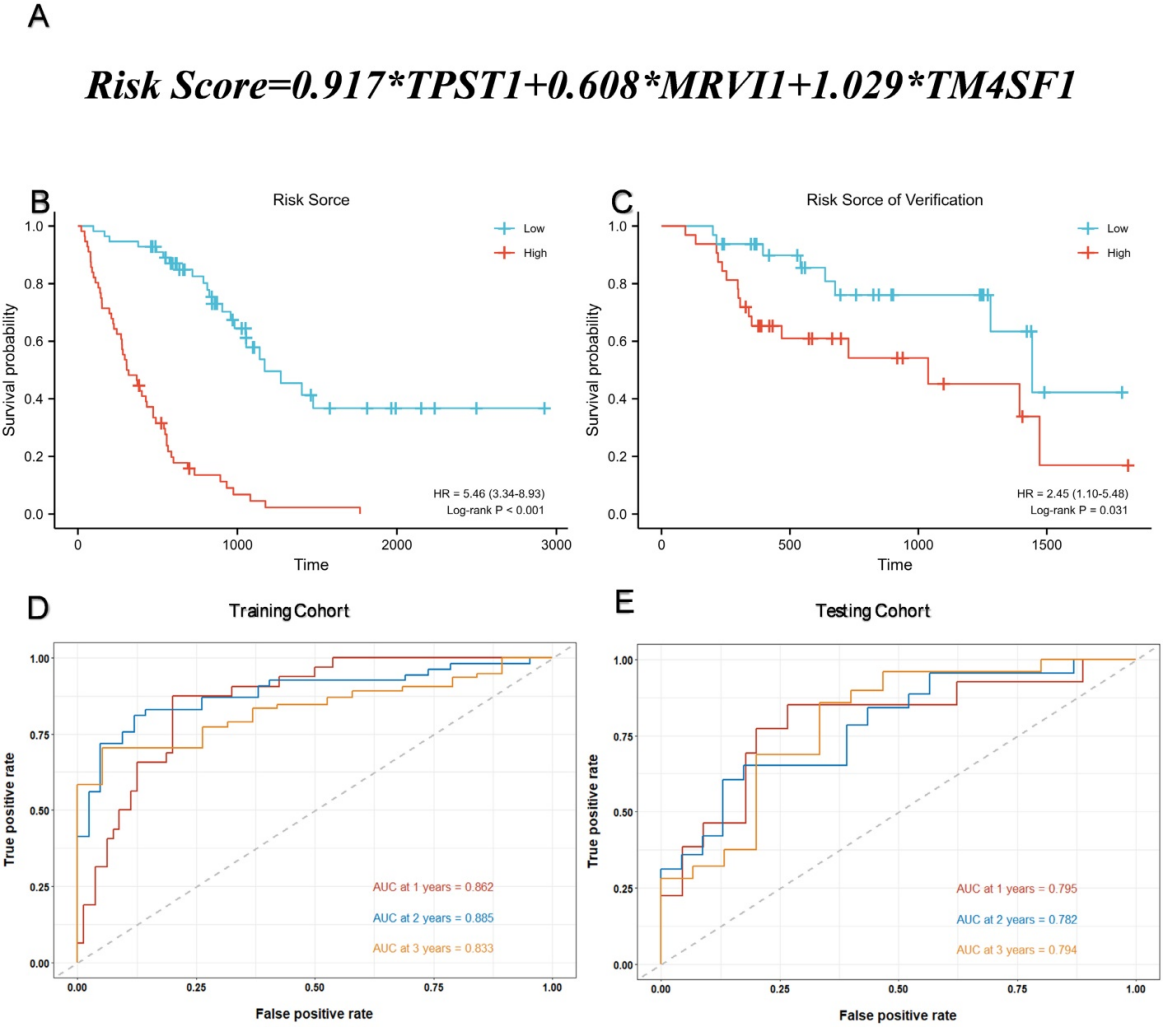
** Assessment of prognostic model performance.** (A) The calculation formula of risk score. (B) Kaplan-Meier survival curve based on the median risk score in the training cohort. (C) Kaplan-Meier survival curve based on the median risk score in the testing cohort. (D) Time-dependent ROC curve for training cohort according to the prognostic model. (E) Time-dependent ROC curve for testing cohort according to the prognostic model. ROC, receiver operator characteristic; AUC, area under the curve.

Multivariate Cox regression was used to calculate the corresponding regression coefficients for TPST1, MRVI1, and TM4SF1, and the risk model was established accordingly. Figure 4(A) shows the formula for calculating the risk score. The 112 patients in the training cohort and 64 patients in the testing cohort were divided into high- and low-risk groups, based on the median risk score. KM survival curves in both cohorts showed that patients in the high-risk group had a more ominous prognosis than those in the low-risk group (Figure 4B-C). Time-dependent ROC curves were generated to detect the accuracy of prediction of the one-, two-, and three-year survival rates in both cohorts. The areas under the curve (AUC) were 0.862, 0.885, and 0.833, respectively, in the training cohort and, AUC in the testing cohort were 0.795, 0.782, and 0.794, respectively (Figure 4D-E).

### Functional annotation of prognosis-associated DEGs

**Figure 5:**
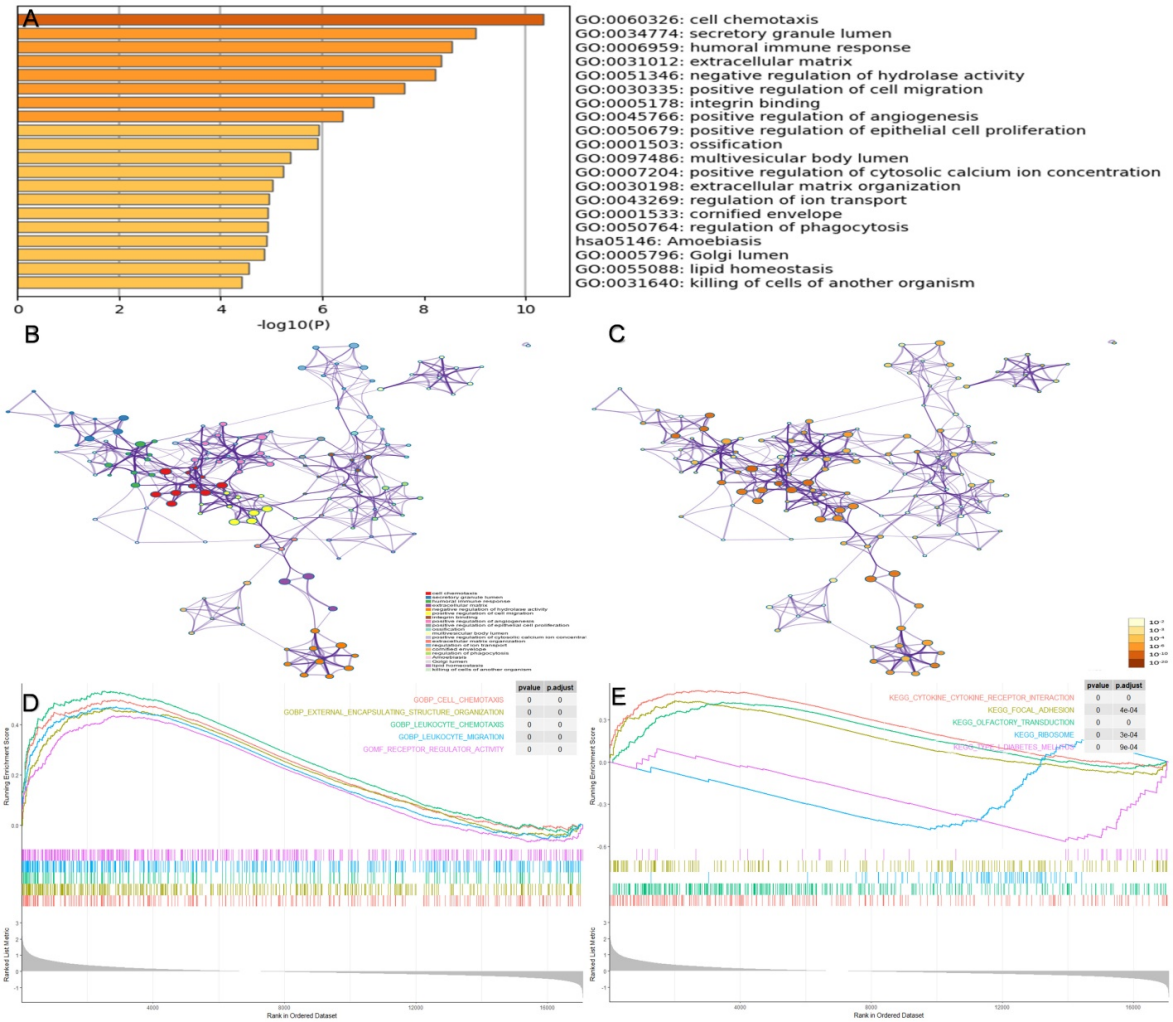
** Enrichment analysis of prognosis-associated DEGs.** (A) The top 20 prognosis-related pathways were enriched based on the Metascape database. (B) The network layout of representative pathways from the cluster. Different clusters were represented by different colors, and the size of nodes was consistent with the proportion of genes in each term. (C) The color of the node is represented by p-value in the same network layout. (D) The top five GO pathways were enriched in high-risk groups via GSEA. (E) The top five KEGG pathways were enriched in high-risk groups via GSEA. GSEA, gene set enrichment analysis.

To identify the potential biological pathways of genes in the prognostic modules, the software Metascape was used for enrichment analysis of all 153 prognosis-associated DEGs. Figure 5(A-C) shows the enriched top 20 prognosis-related pathways, and Table 3 shows the specific information of the top five pathways. We found that biological processes, such as the cell chemotaxis (GO:0060326, P < 0.001), secretory granule lumen (GO:0034774, P < 0.001), humoral immune response (GO:0006959, P < 0.001), extracellular matrix ( GO:0031012, P < 0.001), and negative regulation of hydrolase activity (GO:0051346, P < 0.001), were likely to influence disease progression in patients with IPF. GSEA was conducted in the different risk groups (Figure 5D-E). In contrast to the low-risk group, biological processes including cell chemotaxis, leukocyte chemotaxis, and migration were more active in the high-risk group. In addition, the pathways involved in high-risk groups included the cytokine-cytokine receptor interaction, focal adhesion, and ribosome.

### Immune cell infiltration

**Figure 6:**
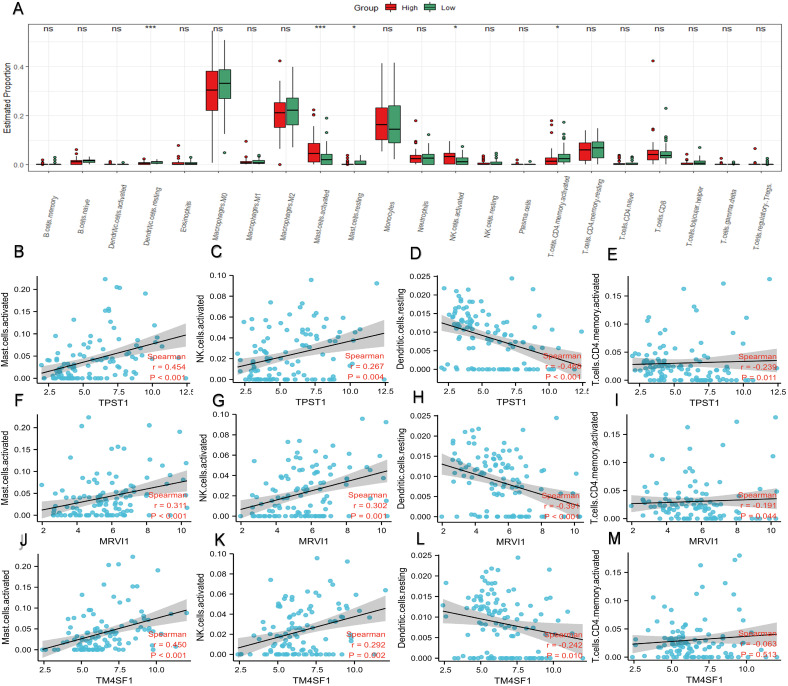
** Profile of immune cell infiltration and its connection to three hub genes.** (A) Difference of immune cell subtypes in different risk groups in the training cohort. The expression levels of TPST1 were correlated with activated mast cell (B), activated NK cell (C), resting dendritic cell (D), and activated CD4+ memory T cells (E). The expression levels of MRVI1 were correlated with activated mast cell (F), activated NK cell (G), resting dendritic cell (H), and activated CD4+ memory T cells (I). The expression levels of TM4SF1 were correlated with activated mast cell (J), activated NK cell (K), resting dendritic cell (L), and activated CD4+ memory T cells (M). *P < 0.05; ***P < 0.001; ns, non-significant.

In the enrichment analysis, we noticed that several pathways, such as humoral immune response, cytokine-cytokine receptor interaction, leukocyte chemotaxis, and migration, were closely related to immune processes. Therefore, we compared the proportions of different types of immune cells in different risk groups (Figure 6A). In contrast to the low-risk group, mast cells and natural killer (NK) cells had higher infiltration degrees in the high-risk group, whereas the infiltration degree of dendritic cells and activated CD4^+^ memory T cells were lower. Spearman correlation analysis was applied to study the association between immune cell subtypes and the expression of the hub genes (Figure 6B-M). The expression of TPST1 was positively associated with activated mast cells (r = 0.454, P < 0.001) but inversely related to resting dendritic cells (r = -0.468, P < 0.001). The expression of MRVI1 was strongly correlated with activated mast cells (r = 0.311, P < 0.001) and activated NK cells (r = 0.302, P < 0.001), and with an increase in MRVI1, the degree of two kinds of cells infiltration was higher. Meanwhile, the expression of TM4SF1 was strongly related to activated mast cells (r = 0.450, P < 0.001) and with an increase in TM4SF1, the degree of infiltration of activated mast cells was higher.

### Construction of the prognostic nomogram

**Figure 7:**
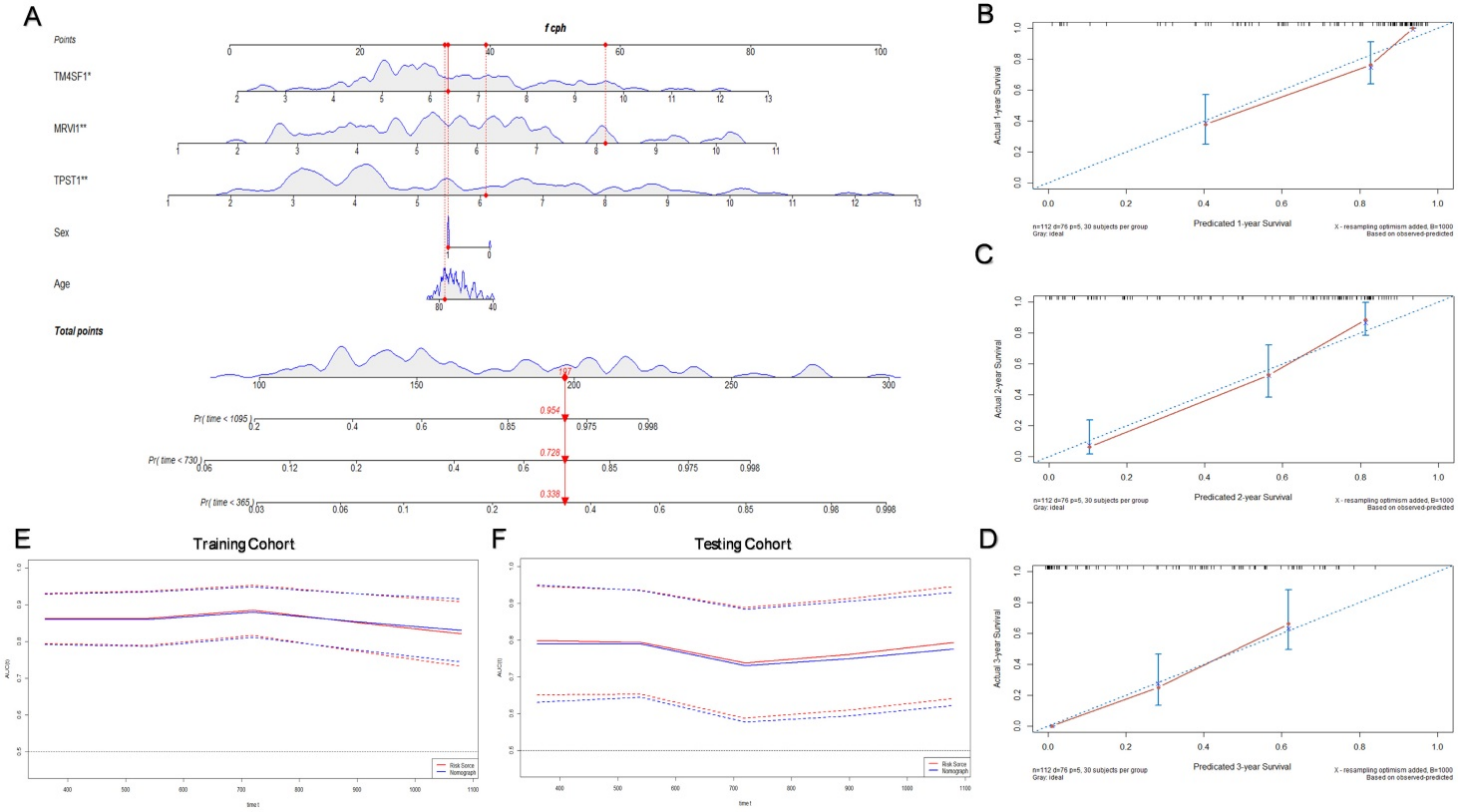
** Construction of the prognostic nomogram.** (A) Nomogram constructed by age, sex, and three hub genes. The calibration curves for prediction of 1- (B), 2- (C), and 3- (D) year survival rate. (E) Comparison of the time-dependent ROC curves for the nomogram and prognostic model in the training cohort. (F) Comparison of the time-dependent ROC curves for the nomogram and prognostic model in the testing cohort.

Considering that the age and sex of patients with IPF can also affect their prognosis, we constructed a nomogram with these significant clinical variables and three hub genes to quantify the 1-, 2-, and 3-year survival probabilities of patients with IPF (Figure 7A). Figure 7(B-D) shows the optimal prediction of the 1-, 2-, and 3-year survival rate based on the calibration curve. Finally, the time-dependent ROC curves of the risk module and nomogram were compared with the prediction efficiency in the training cohort (Figure 7E) and the results were validated in the testing cohort (Figure 7F). In general, there is little difference between the accuracy of two methods in predicting the both short-term and long-term survival rates.

## Discussion

IPF is a chronic respiratory disease with an estimated incidence and prevalence of 0.09-1.30 and 0.33-4.51 cases per 10,000 persons^19^. Because of the short median survival time, many IPF patients experience varying degrees of delay in diagnosis and treatment, leading to poor prognosis^20^. Lamas *et al*. found that the median delay from the onset of clinical symptoms to the first in-hospital diagnosis was approximately 2.2 years, which was associated with a higher rate of death from IPF (P = 0.03)^21^. In the past few years, an increasing number of studies have focused on the cellular and molecular mechanisms of IPF, and progress has been made^22^. Several blood proteins, such as metalloproteases-7 (MMP7), SP-A, and SP-D, can help distinguish IPF from other ILDs, even if they are not included in the guidelines^23^-^25^. In addition, some molecular markers, such as MMPs, tissue inhibitors of MMPs (TIMPs), and alpha-defensins, contribute to the assessment of mechanisms or biological pathways relevant to prognosis^26^, ^27^.

The GSE70866 dataset is derived from the study of BLCs of patients in three independent IPF cohorts by Prasse* et al.* in 2019^13^. In this study, they used nine gene signatures to construct prognostic models and compared the predictive performance of the models in different cohorts. The best prediction of survival time was in the Freiburg cohort (C-index, 0.73; 95% CI, 0.69-0.77), which was still slightly worse than our prediction model (C-index, 0.76; 95% CI, 0.73-0.79). On the one hand, in contrast to using one cohort (62 IPF patients and 20 control individuals) to construct the model by Prasse* et al.*, we combined IPF patients from two cohorts on the basis of eliminating batch differences and standardized data and expanded the sample size in the training set. On the other hand, we used WGCNA to screen hub genes on the basis of differential expression analysis, which narrowed the selection of candidate genes and enhanced their association with survival time. In addition, some studies have used the same dataset to search for molecular markers correlated with the prognosis of IPF. Li *et al*. built a risk model using five ferroptosis-related genes for prognostic prediction in IPF^28^. Xia *et al*. used a method similar to ours to construct a four-gene signature risk model (C-index, 0.72; 95% CI, 0.66-0.77)^29^. However, they used follow-up time as a screening criterion for genes related to prognosis, which reduced the accuracy of their conclusions. In our study, the 1-, 2-, and 3-year AUC values of the time-dependent ROC curve based on the prognostic model were superior to those of previous models (0.862, 0.885, and 0.833 vs 0.737, 0.772, and 0.731 by Xia; 0.773, 0.774, and 0.752 by Li), thus demonstrating the potential applicability of our results.

The results of enrichment analysis indicated that the inflammatory response (such as cell chemotaxis and migration) and immune cell infiltration were key pathways affecting the disease progression of IPF patients and that biological processes mainly occurred in the extracellular matrix. Most pulmonary lung diseases are accompanied by persistent inflammation of the respiratory tract^30^. Alveolar and bronchial epithelial cells damaged by fibrosis release inflammatory mediators and chemokines, which trigger the formation of a provisional ECM and attract inflammatory cells to infiltrate lung tissues. Meanwhile, metalloproteinases and proinflammatory cytokines secreted by inflammatory cells also aggravate apoptosis and lung inflammation^31^. During this process, profibrotic cytokines and growth factors are secreted by activated lymphocytes and other cells; subsequently, fibroblasts are activated. Prolonged inflammatory stimulation leads to excessive accumulation of EMC components, exacerbating the progression of pulmonary fibrosis^32^.

In recent years, a growing body of research has confirmed that innate and adaptive immune mechanisms play a necessary role in the progression of IPF, as confirmed in our study^32^. Neutrophils and macrophages are the primary immune cells in the pathogenesis of IPF, as reported in the literature^33^-^35^. Surprisingly, the results suggest that mast cells may also play an important role in the prognosis of IPF. Research by Veerappan *et al*. has suggested that the deficiency of mast cells in bleomycin-induced mouse models alleviates the decline in lung compliance, and significant fibrosis appears after transplantation of mast cells^36^. In general, the concentrations of histamine and trypsin secreted by mast cells are significantly increased in the BALF of IPF patients, which promotes fibroblast proliferation and synthesis of collagen fibers^33^, ^37^. However, the exact mechanism of mast cell and IPF progression remains to be explored.

In our study, three hub genes (TPST1, MRVI1, and TM4SF1) were identified and found to be related to the prognosis of IPF. MRVI1, also known as IRAG, is the substrate of Inositol 1,4,5-triphosphate receptor-associated cGMP kinase, regulating intracellular IP3- induced calcium release through a NO/PRKG1-dependent mechanism^38^, ^39^. As reported, MRVI1 plays a key role in the progression of some types of cancer, such as endometrial carcinoma, ovarian carcinoma, neurofibromatosis, and involved in platelet activation and aggregatio^n40-42^. Furthermore, activation of calcium channels in mast cells and fibroblasts are closely implicated to the progression of IPF^43^ and MRVI1 is likely to be a key factor affecting this process.

TM4SF1, a tumor-associated protein, is widely expressed in multiple human carcinomas, is localized at the surface of the cell membrane and late endocytic organelles, and plays a vital role in cell motility^44^, ^45^. CD63 is an important activation marker for mast cells and is highly expressed on the surface of activated mast cells^46^. Several experiments have demonstrated that the expression of CD63 is inversely correlated with the motility of various cell types, while the overexpression of TM4SF1 can decrease the expression levels of CD63 on the cell surface, suggesting a potential mechanism for the activation and migration of mast cells^45^, ^47^. In addition, the expression of TM4SF1 in different tumors has different prognostic significances. For example, high expression of TM4SF1 in pancreatic ductal adenocarcinoma indicates a good prognosis, whereas it indicates a poor prognosis in lung cancer^48^, ^49^. The results of our study showed that in the lung tissues of IPF patients, high expression of TM4SF1 may lead to a poor prognosis by guiding mast cell migration and exacerbating the inflammatory process.

Protein-tyrosine sulfotransferase 1(TPST1), a member of the protein sulfotransferase family, catalyzes the sulfuration of tyrosine residues within the acidic motif of polypeptides^50^. Although the specific catalytic mechanism of the TPST1-induced tyrosine sulfation reaction is still unclear, it has been shown that TPST1 regulates immune and inflammatory responses by catalyzing the sulfation of tyrosine residues in some inflammatory mediators, such as chemokine receptors (i.e., CXCR4) and complement (i.e., C5a)^50^, ^51^_._ TPST1 has been reported to be overexpressed in bladder cancer and nasopharyngeal carcinoma^52^, ^53^. Our study showed that TPST1 was highly expressed in the BLAF of patients with IPF and was closely related to inflammatory cell infiltration (mast cells), suggesting that it could affect the prognosis of IPF.

Our study has some limitations. First, the sample size of the GSE70866 dataset was less than 200, while it had a larger sample size and more comprehensive clinical characteristics than most IPF datasets. Second, our study was limited to the inside of the dataset and lacked an effective external verification. Finally, the results of our study were obtained by the analysis of bioinformatics technology, and the specific mechanism by which hub genes affect the prognosis of IPF still needs to be confirmed by further experiments.

## Conclusion

In summary, our study identified three hub genes (TPST1, MRVI1 and TM4SF1) in BLCs that are closely associated with the prognosis of IPF and created a new prognostic model. The results of our study revealed that inflammation and immune processes were the main pathways affecting the prognosis of IPF, and that there was a close relationship between the three hub genes and infiltration of mast cells. Our findings can provide guidance for the prognosis and treatment of patients with IPF.

## Figures and Tables

**Table 1:** The demographic data of training and testing cohorts

	Training cohort		Testing cohort
	Normal	IPF	IPF
N	20	112	64
Age(years)	61.9±7.6	68.0±10.0	68.3±8.5
FVC predicted value (%)	96±19	66.4±21.3	78±18
Sex (%)			
Male	16(80)	93(83)	51(80)
Female	4(20)	19(17)	13(20)
Time to death (days)	NA	698.1±553.4	701.4±451.3
Survival status (%)			
alive	NA	36(32)	40(63)
death	NA	76(68)	24(37)

Data are presented as mean ± SD; FVC stands for forced lung capacity.

**Table 2:** 38 genes associated with prognosis in the turquoise and brown modules

Gene Symbol	Module Color	GS.OS	p.GS.OS	MM	p.MM
ADM	blue	-0.47864	2.41E-07	0.923084	1.53E-44
CCL2	blue	-0.5694	2.32E-10	0.860735	5.69E-32
HTRA1	blue	-0.50464	4.04E-08	0.849588	2.23E-30
S100A12	blue	-0.44701	1.75E-06	0.846673	5.54E-30
HAMP	blue	-0.41311	1.19E-05	0.844997	9.28E-30
PLA2G7	blue	-0.50116	5.17E-08	0.844173	1.19E-29
IER3	blue	-0.44526	1.94E-06	0.840448	3.64E-29
FAM20A	blue	-0.52378	9.83E-09	0.835115	1.72E-28
RGL1	blue	-0.46221	6.92E-07	0.834371	2.12E-28
CCL7	blue	-0.51522	1.87E-08	0.82302	4.74E-27
DYSF	blue	-0.41709	9.59E-06	0.819783	1.1E-26
FAM198B	blue	-0.44442	2.04E-06	0.815487	3.31E-26
MERTK	blue	-0.54821	1.42E-09	0.813605	5.3E-26
CXCL1	blue	-0.43614	3.31E-06	0.81167	8.56E-26
IL1R2	blue	-0.46862	4.62E-07	0.811009	1.01E-25
TPST1	blue	-0.53923	2.94E-09	0.801859	8.95E-25
STEAP4	blue	-0.40392	1.93E-05	0.788823	1.67E-23
MRVI1	blue	-0.46954	4.35E-07	0.771104	6.46E-22
STAB1	blue	-0.56304	4.05E-10	0.761484	4.12E-21
SPP1	blue	-0.46175	7.12E-07	0.751431	2.6E-20
ARAP3	blue	-0.40622	1.71E-05	0.744483	8.86E-20
HS3ST1	blue	-0.5059	3.69E-08	0.737415	2.96E-19
MATK	blue	-0.45653	9.84E-07	0.732764	6.4E-19
MMP7	blue	-0.40206	2.12E-05	0.723649	2.78E-18
TM4SF19	blue	-0.51477	1.93E-08	0.713353	1.36E-17
SH3RF1	blue	-0.45904	8.43E-07	0.701761	7.51E-17
CYR61	black	-0.48367	1.72E-07	0.93291	1.73E-47
MUC21	black	-0.42503	6.2E-06	0.896113	4.02E-38
SFTPB	black	-0.50103	5.22E-08	0.888367	1.33E-36
TM4SF1	black	-0.50926	2.9E-08	0.882091	1.89E-35
CEACAM7	black	-0.52107	1.21E-08	0.85679	2.16E-31
SOD3	black	-0.46694	5.14E-07	0.836578	1.13E-28
MUC1	black	-0.41214	1.25E-05	0.817737	1.87E-26
SFN	black	-0.44782	1.67E-06	0.811948	7.99E-26
TUBB3	black	-0.43214	4.16E-06	0.800136	1.33E-24
EMP1	black	-0.50598	3.67E-08	0.770788	6.88E-22
PRSS8	black	-0.48262	1.85E-07	0.745873	6.96E-20
S1PR3	black	-0.44095	2.5E-06	0.730392	9.43E-19

**Table 3:** The top five biological pathways enriched by Metascape database

Category	Term	Description	Log P	Log(q-value)	Symbols
GO Biological Processes	GO:0060326	cell chemotaxis	-10.35512264	-6.002149463	CCR3,GAS6,CXCL1,CXCL8,CCN3,S100A12, CCL2,CCL7,CCL13,SFTPD
GO Cellular Components	GO:0034774	secretory granule lumen	-9.006327538	-5.412083655	CAMP,CDA,DEFA3,ECM1,F13A1,FCN1, FOLR3,GAS6,CXCL1,IGF1
GO Biological Processes	GO:0006959	humoral immune response	-8.538668442	-5.08878525	C8A,C8B,CAMP,DEFA3,FCN1,CXCL1,CXCL8, PI3,S100A12,CCL2,CCL13,SFTPD,CXCL1
GO Cellular Components	GO:0031012	extracellular matrix	-8.3298266	-5.04477331	CLC,ECM1,F13A1,FCN1,CCN1,LAMB1,MMP7, MMP9,MMP10,CCN3,PI3
GO Biological Processes	GO:0051346	negative regulation of hydrolase activity	-8.219075562	-5.012230419	CST6,ECM1,GAS6,SFN,MMP9,PI3, SERPINI2,RGS2,SPINK1,TIMP3

## Abbreviations

adj.P: adjusted P value
AUC: areas under the curve
BAL: bronchoalveolar lavage
BALF: bronchoalveolar lavage fluid
BLCs: bronchoalveolar lavage cells
DEGs: differentially expressed genes
ECM: extracellular matrix
GEO: gene expression omnibus
GS: gene significance
GSEA: gene set enrichment analysis
ILD: interstitial lung disease
IPF: idiopathic pulmonary fibrosis
MM: module members
NK: natural killer
ROC: receiver operator characteristic
WGCNA: weighted gene co-expression network analysis

## References

[1] King TE Jr, Pardo A, Selman M (2011). Idiopathic pulmonary fibrosis. Lancet.

[2] Ruigrok MJR, Frijlink HW, Melgert BN, Olinga P, Hinrichs WLJ (2021). Gene therapy strategies for idiopathic pulmonary fibrosis: recent advances, current challenges, and future directions. Mol Ther Methods Clin Dev.

[3] Richeldi L, Collard HR, Jones MG (2017). Idiopathic pulmonary fibrosis. Lancet.

[4] Martinez FJ, Collard HR, Pardo A, Raghu G, Richeldi L, Selman M (2017). Idiopathic pulmonary fibrosis. Nat Rev Dis Primers.

[5] Sgalla G, Biffi A, Richeldi L (2016). Idiopathic pulmonary fibrosis: Diagnosis, epidemiology and natural history. Respirology.

[6] Martinez FJ, Safrin S, Weycker D, Starko KM, Bradford WZ, King TE Jr (2005). The clinical course of patients with idiopathic pulmonary fibrosis. Ann Intern Med.

[7] Krishna R, Chapman K, Ullah S (2022). Idiopathic Pulmonary Fibrosis. StatPearls. Treasure Island (FL): StatPearls Publishing.

[8] Njock MS, Guiot J, Henket MA, Nivelles O, Thiry M, Dequiedt F (2019). Sputum exosomes: promising biomarkers for idiopathic pulmonary fibrosis. Thorax.

[9] Stainer A, Faverio P, Busnelli S, Catalano M, Della Zoppa M, Marruchella A (2021). Molecular Biomarkers in Idiopathic Pulmonary Fibrosis: State of the Art and Future Directions. Int J Mol Sci.

[10] Patel PH, Antoine M, Ullah S (2022). Bronchoalveolar Lavage. StatPearls. Treasure Island (FL): StatPearls Publishing.

[11] Meyer KC, Raghu G, Baughman RP, Brown KK, Costabel U, du Bois RM (2012). An official American Thoracic Society clinical practice guideline: the clinical utility of bronchoalveolar lavage cellular analysis in interstitial lung disease. Am J Respir Crit Care Med.

[12] Langfelder P, Horvath S (2008). WGCNA: an R package for weighted correlation network analysis. BMC Bioinformatics.

[13] Prasse A, Binder H, Schupp JC, Kayser G, Bargagli E, Jaeger B (2019). BAL Cell Gene Expression Is Indicative of Outcome and Airway Basal Cell Involvement in Idiopathic Pulmonary Fibrosis. Am J Respir Crit Care Med.

[14] Robinson MD, McCarthy DJ, Smyth GK (2010). edgeR: a Bioconductor package for differential expression analysis of digital gene expression data. Bioinformatics.

[15] Simon N, Friedman J, Hastie T, Tibshirani R (2011). Regularization Paths for Cox's Proportional Hazards Model via Coordinate Descent. J Stat Softw.

[16] Zhou Y, Zhou B, Pache L, Chang M, Khodabakhshi AH, Tanaseichuk O (2019). Metascape provides a biologist-oriented resource for the analysis of systems-level datasets. Nat Commun.

[17] Yu G, Wang LG, Han Y, He QY (2012). clusterProfiler: an R package for comparing biological themes among gene clusters. Omics.

[18] Newman AM, Liu CL, Green MR, Gentles AJ, Feng W, Xu Y (2015). Robust enumeration of cell subsets from tissue expression profiles. Nat Methods.

[19] Maher TM, Bendstrup E, Dron L, Langley J, Smith G, Khalid JM (2021). Global incidence and prevalence of idiopathic pulmonary fibrosis. Respir Res.

[20] Glassberg MK (2019). Overview of idiopathic pulmonary fibrosis, evidence-based guidelines, and recent developments in the treatment landscape. Am J Manag Care.

[21] Lamas DJ, Kawut SM, Bagiella E, Philip N, Arcasoy SM, Lederer DJ (2011). Delayed access and survival in idiopathic pulmonary fibrosis: a cohort study. Am J Respir Crit Care Med.

[22] Zhu K, Xu A, Xia W, Li P, Han R, Wang E (2021). Integrated analysis of the molecular mechanisms in idiopathic pulmonary fibrosis. Int J Med Sci.

[23] Bauer Y, White ES, de Bernard S, Cornelisse P, Leconte I, Morganti A (2017). MMP-7 is a predictive biomarker of disease progression in patients with idiopathic pulmonary fibrosis. ERJ Open Res.

[24] Wang K, Ju Q, Cao J, Tang W, Zhang J (2017). Impact of serum SP-A and SP-D levels on comparison and prognosis of idiopathic pulmonary fibrosis: A systematic review and meta-analysis. Medicine (Baltimore).

[25] Zhu C, Zhao YB, Kong LF, Li ZH, Kang J (2016). [The expression and clinical role of KL-6 in serum and BALF of patients with different diffuse interstitial lung diseases]. Zhonghua Jie He He Hu Xi Za Zhi.

[26] Todd JL, Vinisko R, Liu Y, Neely ML, Overton R, Flaherty KR (2020). Circulating matrix metalloproteinases and tissue metalloproteinase inhibitors in patients with idiopathic pulmonary fibrosis in the multicenter IPF-PRO Registry cohort. BMC Pulm Med.

[27] Konishi K, Gibson KF, Lindell KO, Richards TJ, Zhang Y, Dhir R (2009). Gene expression profiles of acute exacerbations of idiopathic pulmonary fibrosis. Am J Respir Crit Care Med.

[28] Li M, Wang K, Zhang Y, Fan M, Li A, Zhou J (2021). Ferroptosis-Related Genes in Bronchoalveolar Lavage Fluid Serves as Prognostic Biomarkers for Idiopathic Pulmonary Fibrosis. Front Med (Lausanne).

[29] Xia Y, Lei C, Yang D, Luo H (2021). Construction and validation of a bronchoalveolar lavage cell-associated gene signature for prognosis prediction in idiopathic pulmonary fibrosis. Int Immunopharmacol.

[30] Racanelli AC, Kikkers SA, Choi AMK, Cloonan SM (2018). Autophagy and inflammation in chronic respiratory disease. Autophagy.

[31] Cabrera S, Maciel M, Herrera I, Nava T, Vergara F, Gaxiola M (2015). Essential role for the ATG4B protease and autophagy in bleomycin-induced pulmonary fibrosis. Autophagy.

[32] Wynn TA (2008). Cellular and molecular mechanisms of fibrosis. J Pathol.

[33] Heukels P, Moor CC, von der Thüsen JH, Wijsenbeek MS, Kool M (2019). Inflammation and immunity in IPF pathogenesis and treatment. Respir Med.

[34] White ES, Lazar MH, Thannickal VJ (2003). Pathogenetic mechanisms in usual interstitial pneumonia/idiopathic pulmonary fibrosis. J Pathol.

[35] Shenderov K, Collins SL, Powell JD, Horton MR (2021). Immune dysregulation as a driver of idiopathic pulmonary fibrosis. J Clin Invest.

[36] Veerappan A, O'Connor NJ, Brazin J, Reid AC, Jung A, McGee D (2013). Mast cells: a pivotal role in pulmonary fibrosis. DNA Cell Biol.

[37] Walls AF, Bennett AR, Godfrey RC, Holgate ST, Church MK (1991). Mast cell tryptase and histamine concentrations in bronchoalveolar lavage fluid from patients with interstitial lung disease. Clin Sci (Lond).

[38] Oberheim Bush NA, Nedergaard M (2017). Do Evolutionary Changes in Astrocytes Contribute to the Computational Power of the Hominid Brain?. Neurochem Res.

[39] Schlossmann J, Desch M (2011). IRAG and novel PKG targeting in the cardiovascular system. Am J Physiol Heart Circ Physiol.

[40] Zhou Z, Xu YP, Wang LJ, Kong Y (2019). miR-940 potentially promotes proliferation and metastasis of endometrial carcinoma through regulation of MRVI1. Biosci Rep.

[41] Kim YS, Hwan JD, Bae S, Bae DH, Shick WA (2010). Identification of differentially expressed genes using an annealing control primer system in stage III serous ovarian carcinoma. BMC Cancer.

[42] Santoro C, Giugliano T, Kraemer M, Torella A, Schwitalla JC, Cirillo M (2018). Whole exome sequencing identifies MRVI1 as a susceptibility gene for moyamoya syndrome in neurofibromatosis type 1. PLoS One.

[43] Roach KM, Bradding P (2020). Ca(2+) signalling in fibroblasts and the therapeutic potential of K(Ca)3.1 channel blockers in fibrotic diseases. Br J Pharmacol.

[44] Fu F, Yang X, Zheng M, Zhao Q, Zhang K, Li Z (2020). Role of Transmembrane 4 L Six Family 1 in the Development and Progression of Cancer. Front Mol Biosci.

[45] Lekishvili T, Fromm E, Mujoomdar M, Berditchevski F (2008). The tumour-associated antigen L6 (L6-Ag) is recruited to the tetraspanin-enriched microdomains: implication for tumour cell motility. J Cell Sci.

[46] Orinska Z, Hagemann PM, Halova I, Draber P (2020). Tetraspanins in the regulation of mast cell function. Med Microbiol Immunol.

[47] Pols MS, Klumperman J (2009). Trafficking and function of the tetraspanin CD63. Exp Cell Res.

[48] Zheng B, Ohuchida K, Cui L, Zhao M, Shindo K, Fujiwara K (2015). TM4SF1 as a prognostic marker of pancreatic ductal adenocarcinoma is involved in migration and invasion of cancer cells. Int J Oncol.

[49] Kao YR, Shih JY, Wen WC, Ko YP, Chen BM, Chan YL (2003). Tumor-associated antigen L6 and the invasion of human lung cancer cells. Clin Cancer Res.

[50] Ippel JH, de Haas CJ, Bunschoten A, van Strijp JA, Kruijtzer JA, Liskamp RM (2009). Structure of the tyrosine-sulfated C5a receptor N terminus in complex with chemotaxis inhibitory protein of Staphylococcus aureus. J Biol Chem.

[51] Seibert C, Veldkamp CT, Peterson FC, Chait BT, Volkman BF, Sakmar TP (2008). Sequential tyrosine sulfation of CXCR4 by tyrosylprotein sulfotransferases. Biochemistry.

[52] Chen Z, Liu G, Hossain A, Danilova IG, Bolkov MA, Liu G (2019). A co-expression network for differentially expressed genes in bladder cancer and a risk score model for predicting survival. Hereditas.

[53] Xu J, Deng X, Tang M, Li L, Xiao L, Yang L (2013). Tyrosylprotein sulfotransferase-1 and tyrosine sulfation of chemokine receptor 4 are induced by Epstein-Barr virus encoded latent membrane protein 1 and associated with the metastatic potential of human nasopharyngeal carcinoma. PLoS One.

